# Combining temperate fruit tree cultivars to fit spring phenology models

**DOI:** 10.1007/s00484-025-03068-2

**Published:** 2026-01-21

**Authors:** Lars Caspersen, Katja Schiffers, Katherine Jarvis-Shean, Eike Luedeling

**Affiliations:** 1 Department of Horticultural Sciences, Institute of Crop Science and Resource Conservation (INRES), University of Bonn, Auf dem Hügel 6, Bonn, 53121 Germany; 2 Division of Agriculture and Natural Resources, University of California, 70 Cottonwood St, Woodland, CA 95695 USA

**Keywords:** Dormancy, Model calibration, Data-scarcity, Almond, Phenology, Flowering

## Abstract

Phenological datasets for temperate fruit trees are often short, fragmented and geographically restricted, which hampers the development of cultivar-specific spring phenology models. To address this, we propose a novel calibration approach (“combined-fitting”), which pools observations from several cultivars of the same species, distinguishing between shared and cultivar-specific parameters. This method requires fewer observations per cultivar and allows jointly analyzing cultivars of the same species. We evaluate combined-fitting using the PhenoFlex framework, comparing it to a baseline model and to models that are fitted only with data for single cultivars (“cultivar-fit”). Our analysis is based on flowering data from nine almond, six apricot and six sweet cherry cultivars across Mediterranean (Spain, Morocco, Tunisia) and German climates. The combined-fit model failed to achieve higher prediction accuracy compared to the cultivar-fit and the baseline approach, as evidenced by similar root mean square errors across the data splits and calibration dataset sizes. When comparing the estimated parameters of the chill and heat accumulation submodels, we observed a large variation among cultivars of the same species in the cultivar-fit models. In contrast and by design, the combined-fit yielded only one parameter set for cultivars of the same species. Our findings demonstrate that integrating data from multiple cultivars can yield spring phenology models with high accuracy. Even though the combined-fit approach did not outperform the cultivar-fit approach, combined-fitting offers a practical solution for spring phenology modeling with limited datasets and facilitates comparison across cultivars of the same species.

**Supplementary Information:**

The online version contains supplementary material available at 10.1007/s00484-025-03068-2.

## Introduction

Forecasting spring phenology (e.g. budburst, flowering) of temperate fruit trees has gained significant attention in plant physiology, horticultural science and climate change research (Bartolini et al. 2019; Caspersen et al. 2025a, b; Chuine et al. 2016; Orozco et al. 2024). For temperate tree species, bloom timing is influenced not only by spring temperatures but also by agroclimatic conditions during winter. Flower buds require exposure to cool temperatures (winter chill) to become responsive to the subsequent warm conditions (heat or forcing) that trigger dormancy release and initiate budbreak (Heide and Prestrud 2005). In warm growing regions, where fruit and nut trees already face a risk of insufficient chilling, continued warming could lead to delayed flowering or even failure to flower normally (Caspersen et al. 2025b; Fernandez et al. 2023; Luedeling and Brown 2011).

It is well established that temperate fruit tree species and cultivars have distinct chill and heat requirements, but reliably quantifying these requirements remains challenging. Numerous models have been proposed to estimate chill and heat accumulation, yet many of them yield requirement estimates that are only valid for the specific climate in which they were developed and fail to generalize across different climatic conditions (Fernandez et al. 2020; Luedeling and Brown 2011). The Dynamic Model (Fishman et al. 1987a, b) for chill accumulation is considered to be particularly robust, especially in warmer climates (Fernandez et al. 2020; Luedeling and Brown 2011). There is less diversity in models quantifying heat accumulation, with the Growing Degree Hours model (Anderson et al. 1986) being the most widely used. Some studies have suggested that accumulated chill and heat needed to overcome dormancy may, to some extent, compensate for each other (Kaufmann and Blanke 2019; Okie and Blackburn 2011), and varying degrees of overlap between chill and heat accumulation have been reported (Pope et al. 2014).

Even when phenologists agree on which models to use for chill and heat accumulation, they often differ in how they define the requirements and how they represent the interaction between chill and heat accumulation. In studies based on experiments with cuttings of young branches, the endodormancy release date is typically identified as the point when a certain proportion (e.g. 50%) of buds break after branches are moved from field to greenhouse conditions at various points during the dormancy season (Ruiz et al. 2007). Once this date is determined, chill requirements are estimated as the accumulated chill up to the end of the endodormancy period. However, cutting experiments across different climatic conditions for the same cultivars lead to inconsistent estimates of chill and heat requirements, even when studies follow a standardized protocol (Delgado et al. 2026). Studies based on Partial Least Squares (PLS) regression aim to identify the time windows during which variation in chill and heat accumulation are associated with variation in bloom date (Luedeling and Gassner 2012). After identifying the relevant chill and heat periods, the requirements are estimated as mean chill and heat accumulated during these periods. This can result in a range of interaction patterns between chill and heat accumulation within these windows, from distinct phases (Benmoussa et al. 2017) to partially overlapping phases (Martínez-Lüscher et al. 2017). Spring phenology models also vary in their structural assumptions: some assume sequential chill and heat accumulation, or parallel accumulation (Chuine 2000; Legave et al. 2013), while some impose a fixed degree of overlap between the two phases (Pope et al. 2014). Despite these differences, most models rely on standard chill and heat submodel parameters. This has the advantage of reducing the numbers of parameters to estimate and facilitate comparison across species and cultivars. However, models tailored to specific species or cultivars may offer a more accurate representation of dormancy release dynamics.

The chill and heat submodels currently used in spring phenology models were originally developed for specific cases rather than for broad application across diverse temperate tree species. For instance, the development of the Dynamic Model was based on controlled temperature experiments in peach trees (Fishman et al. 1987a, b). Research suggests that adjusting the parameters of the Dynamic Model for specific cultivars or species can reduce year-to-year variability in estimated chill requirements (Egea et al. 2021). Consequently, models like PhenoFlex (Luedeling et al. 2021) propose estimating not only chill and heat requirements but also parameters governing chill and heat dynamics, including potential interactions between the two. The underlying principle is that, while the need for both chill and heat is generally shared across species, different species and cultivars may have distinct optimal temperature ranges for chill and heat accumulation. Several studies have demonstrated the effectiveness of models for which the chill and heat accumulation submodels were tailored for specific cultivars (Caspersen et al. 2024, 2025b; Fernandez et al. 2022; Luedeling et al. 2021). The primary advantage of this approach is that it optimizes submodels based on available data, but a major drawback is that it increases the number of parameters requiring calibration, potentially demanding a larger dataset for accurate model fitting.

Customizing chill and heat submodels, as proposed in the PhenoFlex framework, may offer advantages over models that rely solely on default parameters. However, the development of specific chill and heat submodels for each cultivar would lead to a proliferation of cultivar-specific models that would be difficult to compare. It would also restrict phenology modeling to cultivars for which abundant phenology observations are available. We propose a pragmatic middle-ground strategy for phenology modeling, in which chill and heat submodels are fitted at the species level while chill and heat requirements are fitted specifically for each cultivar. This strategy allows pooling observations from multiple cultivars of the same species, including data for cultivars that, on their own, have not been covered by sufficient observations for full cultivar-level calibration. The relevance of this benefit is illustrated by a multi-species dataset of long-term phenology records that was recently published by Luedeling et al. (2024a). Out of nearly 270 tree cultivars contained in the dataset, only 110 had sufficient data for cultivar-specific model-fitting (Caspersen et al., 2025b). Many cultivars had to be dropped from the analysis because they did not have at least 20 phenological observations, as required according to the vignette accompanying PhenoFlex (Urbach et al. 2021).

In this study, we propose a new calibration method for phenology models tailored to situations where individual cultivars have limited observations, but complementary data from other locations or related cultivars are available. Our approach assumes that while chill and heat requirements, as well as the interactions between chill and heat accumulation, vary among cultivars, the fundamental mechanisms governing chill and heat accumulation remain consistent.

We evaluate this newly proposed method alongside cultivar-level calibration and a baseline approach using default parameters for chill and heat submodels for three temperate fruit tree species: almond (*Prunus dulcis* D.A.), apricot (*Prunus armeniaca* L.) and sweet cherry (*Prunus avium* L.). Our analysis assesses model performance under both data-rich and data-scarce conditions. Additionally, we investigate the sensitivity of calibrated chill and heat submodels to different temperatures by generating temperature response curves. The bloom time dataset comprises observations from southern Spain, Morocco and Tunisia for almonds; northern and southern Spain for apricots; and northern Spain and Germany for sweet cherries.

## Materials and methods

### Phenological data

**Fig. 1:**
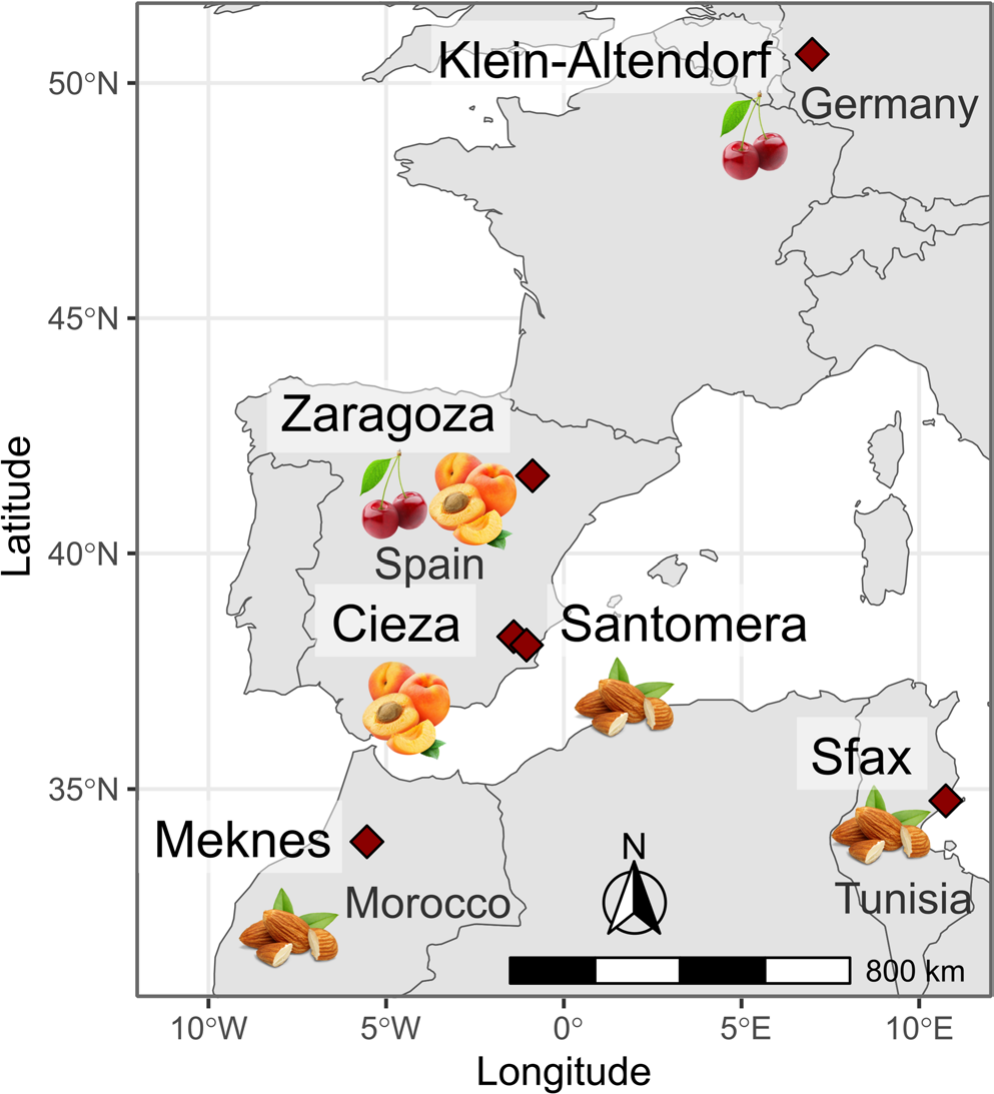
Overview of the locations with bloom records. The species for which data were collected in each location are indicated by pictures below the location name

We analyzed full bloom observation data of three species – almond, apricot and sweet cherry – across 21 cultivars from the Mediterranean region and Germany (Luedeling et al., 2024a). The data are part of a publicly available repository (10.60507/FK2/MZIELI). Full bloom corresponds to stage 65 on the BBCH scale (Biologische Bundesanstalt für Land- und Forstwirtschaft, Bundessortenamt, Chemische Industrie), which is defined as 50% of flower buds having opened (Meier 2018). Phenological observations were collected in various locations: almonds in Santomera (southern Spain), Meknes (Morocco) and Sfax (Tunisa); apricots in Cieza (southern Spain) and Zaragoza (northern Spain); and sweet cherries in Zaragoza (northern Spain) and Klein-Altendorf (Germany) (Fig. 1; Table 1). The observation periods varied by location, with the earliest records dating back to 1974 and extending up to 2022. A previous study analyzed parts of this dataset using PhenoFlex to project climate change impacts (Caspersen et al. 2025b). A comprehensive summary of the phenological data is provided in Online Resource 1, Table S1.

**Table 1:** Overview of the bloom observations per species and location, including the year with earliest and latest observations and the total number of bloom observations

Species	Location	Country	Coordinates (latitude; longitude)	Cultivars	Covered period	Total number of observations
Almond	Meknes	Morocco	33.88°; 5.54°	Ferragnes, Marcona, Tuono	1974–2014	117
	Santomera	Spain	38.06°; −1.05°	Achaak, Desmayo, Marta	1997–2022	48
	Sfax	Tunisia	34.75°; 10.75°	Fasciuneddu, Mazzetto, Nonpareil	1981–2016	67
Apricot	Cieza	Spain	38.24°; −1.41°	Búlida, Dorada	2002–2022	41
	Zaragoza	Spain	41.65°; −0.88°	Goldrich, Harcot, Henderson, Sunglo	1999–2022	86
Sweet Cherry	Klein-Altendorf	Germany	50.61°; 6.99°	Burlat, Regina, Schneiders	1984–2020	93
	Zaragoza	Spain	41.65°; −0.88°	Rainier, Sam, Van	1991–2022	72

### Local temperature data

We collected daily minimum and maximum temperatures from local weather stations in the orchards and publicly available weather stations in the Global Summary of the Day (GSOD) database. To address missing data, we used observations from nearby weather stations. Before gap-filling, we applied bias correction by calculating the mean temperature difference between stations over a shared observation period, using a 15-day moving window centered on the missing-data day. We corrected the observation of the auxiliary station by subtracting the calculated mean difference before filling the gap. The calculated mean biases between target and auxiliary stations did not exceed ± 3 °C and the standard deviation was below 3 °C. Auxiliary stations were located within 70 km of the target station. When no suitable auxiliary data were available, we applied linear interpolation, though this was necessary for less than 2% of the total observation period. Further details on temperature data processing are available in Caspersen et al. (2025b).

### Phenology model

We used the PhenoFlex phenology modeling framework (Luedeling et al. 2021), which has performed well in predicting tree bloom under field and experimental conditions (Caspersen et al. 2025b; Fernandez et al. 2022; Luedeling et al. 2021; Picornell et al. 2023). PhenoFlex integrates two submodels: the Dynamic Model (Fishman et al. 1987a, b) for chill accumulation and the Growing Degree Hours (GDH) model (Anderson et al. 1986) for heat accumulation.

Chill accumulation is controlled by six parameters (E_0_, E_1_, A_0_, A_1_, T_f_, slope), while heat accumulation depends on three parameters (T_b_, T_u_, T_c_; Table 2). Additionally, PhenoFlex introduces three parameters: y_c_ (chill requirement), z_c_, (heat requirement), and s_1_ (transition from chill to heat accumulation). As accumulated chill nears the chill requirement (y_c_), the potential for heat accumulation rises. A higher s_1_ value promotes sequential chill and heat accumulation, while lower values result in more parallel accumulation.

The model predicts bloom as the earliest day when accumulated heat meets the heat requirement (z_c_). If heat requirement remains insufficient by the end of the time series, the model records a failure to bloom.

### Calibration experiments

We calibrated PhenoFlex for nine almond cultivars, six apricot cultivars and six sweet cherry cultivars (Table 1). We selected sets of cultivars that covered the whole range of bloom timings across locations and species.

**Fig. 2:**
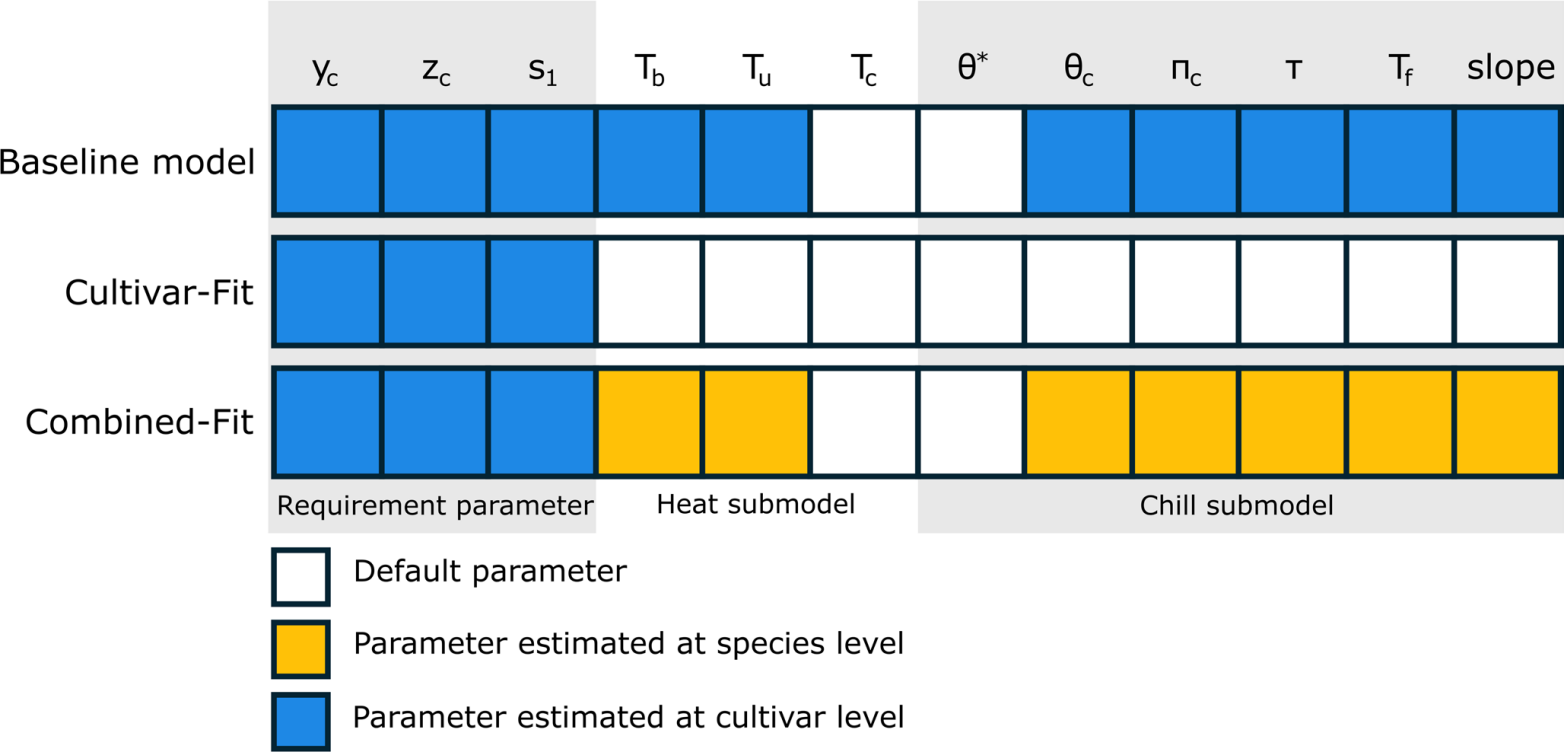
Schematic representation of calibration approaches for *PhenoFlex*: “Baseline model” with default parameters of chill and heat sub-models (white boxes) and only calibration of cultivar-specific requirement parameters (blue boxes); “Cultivar-fit” model with calibration of all model parameters for each cultivar separately; “Combined-fit” model with species-specific calibration of heat and chill sub-models (yellow boxes) but cultivar-specific requirement parameters (blue boxes). Parameter θ^*^ (chill sub-model) and T_c_ (heat sub-model) are kept constant as θ^*^ = 279 K and T_c_ = 36 °C throughout the analysis

We split the data into a calibration and a validation subset. We used the calibration data to estimate the model parameters, while the validation dataset was used to evaluate model performance. We employed two data splits: (1) full calibration (75% calibration “Full_cal_”, 25% validation “Full_val_”, leading to 10 to 30 observations per cultivar for calibration), and (2) scarce calibration (10 observation per cultivar for calibration “Scarce_cal_” and remaining data in validation “Scarce_val_”). We tested three calibration approaches (Fig. 2):

1. **Baseline model**: Standard chill and heat submodel parameters, (chill accumulation: E_0_ = 3372.8e + 13, E_1_ = 9900.3, A_0_ = 6319.5, A_1_ = 5.939917e + 13, T_f_ = 4 °C, slope = 1.6; heat accumulation: T_b_ = 4 °C, T_u_ = 26 °C, T_c_ = 36 °C). Only the requirement parameters y_c_, z_c_, s_1_ were optimized for each cultivar.
2. **Cultivar-fit**: Independent chill and heat submodels for each cultivar, using fixed values for two of the twelve model parameters (T_c_ = 36 °C and θ^*^ = 279 K), which have been shown to have little impact on model performance (Caspersen et al. 2024).
3. **Combined-fit**: Cultivar-specific requirement parameters (y_c_, z_c_, s_1_) and species-wide chill and heat submodel parameters, reducing the number of cultivar-specific parameters from 10 to three.

For model calibration, we used the R package MEIGO to apply two fitting algorithms: Enhanced Scatter Search and Dynamic Hill Climbing (Egea et al. 2014). We substituted a subset of chill accumulation parameters (E_0_, E_1_, A_0_, and A_1_) with more narrowly defined intermediate parameters (θ^*^, θ_c_, τ and π_c_). Following the approach outlined by Egea et al. (2021) and applied to the PhenoFlex model by Caspersen et al. 2024, 2025b), we converted the intermediate parameters into the original parameter set before running the model. More details on the parameter search space and optimization settings are given in Table 2 and Online Resource 1.

**Table 2:** Model parameters and their upper and lower ranges during the optimization process

Parameter	Name	Unit	Lower limit	Upper limit
y_c_	Chill requirement	Variant of chill portions	5	80
z_c_	Heat requirement	Variant of Growing Degree Hours	100	700
s_1_	Slope of transition from chill to heat accumulation	No unit	0.1	1.2
θ^*^	Optimal constant temperature for chill accumulation	K	Kept constant at 279 K	
θ_c_	Critical constant temperature above which no more chill accumulation takes place	K	286	287
τ	Time interval for accumulating one chill portion at optimal temperature	h	16	48
π_c_	Critical period in a combined temperature cycle leading to chill negation	h	24	50
E_0_	Time-independent activation energy for forming PDBF	K	Calculated based on intermediate parameters (θ^*^, θ_c_, τ, π_c_)	
E_1_	Time-independent activation energy for destroying PDBF	K	Calculated based on intermediate parameters (θ^*^, θ_c_, τ, π_c_)	
A_0_	Amplitude of the process involved in forming PDBF	1/h	Calculated based on intermediate parameters (θ^*^, θ_c_, τ, π_c_)	
A_1_	Amplitude of the process involved in destroying PDBF	1/h	Calculated based on intermediate parameters (θ^*^, θ_c_, τ, π_c_)	
T_f_	Transition temperature for conversion of PDBF to DBF	°C	2	10
Slope	Slope of transition from PDBF to DBF	No unit	1.2	5.0
T_b_	Base temperature for heat accumulation	°C	0	10
T_u_	Optimal temperature for heat accumulation	°C	15	30
T_c_	Critical temperature for heat accumulation	°C	Kept constant at 36 °C	

### Validation

We evaluated the calibrated models using the withheld observations. We calculated Root Mean Square Error (RMSE), Ratio of Performance to Interquartile Distance (RPIQ) and mean bias (predicted minus observed dates) for calibration and validation data. Additionally, we calculated temperature response curves for the estimated chill and heat submodels. The temperature response curves illustrate the chilling and forcing processes characterized by the fitted parameters. We applied long periods (1200 h) of constant temperatures ranging from − 5 °C to 50 °C (at intervals of 0.1 °C) to the sub models and recorded the model outputs that resulted from these inputs.

### Technical information

All analyses were conducted using R (R Core Team 2022) and RStudio (Posit team 2024) version 2024.4.0.735. For climate data processing and phenological modeling, we used the chillR package, version 0.75 (Luedeling et al. 2024b). Adjusted PhenoFlex evaluation functions for baseline model and combined-fitting were implemented in the evalpheno (Caspersen 2025a) and LarsChill (Caspersen 2025b) packages. Model calibration relied on MEIGOR (Egea et al. 2014). For data handling and visualization, we used the tidyverse framework, version 1.3.2 (Wickham et al. 2019). Calibration experiments were run partly on the “bonna” computing cluster. A link to the code for the analysis can be found in the Online Resource 1 and via: https://larscaspersen.github.io/combined-fitting-bloom/.

## Results

### Validating the calibrated models

We compared three methods for calibrating the PhenoFlex spring phenology framework: (i) a baseline model with standard chill and heat submodels coupled with cultivar-specific requirement parameters, (ii) cultivar-fit models with the entire models calibrated for each cultivar individually and (iii) combined-fit models with jointly calibrated chill and heat sub-models across cultivars of the same species but cultivar-specific requirement parameters.

**Fig. 3:**
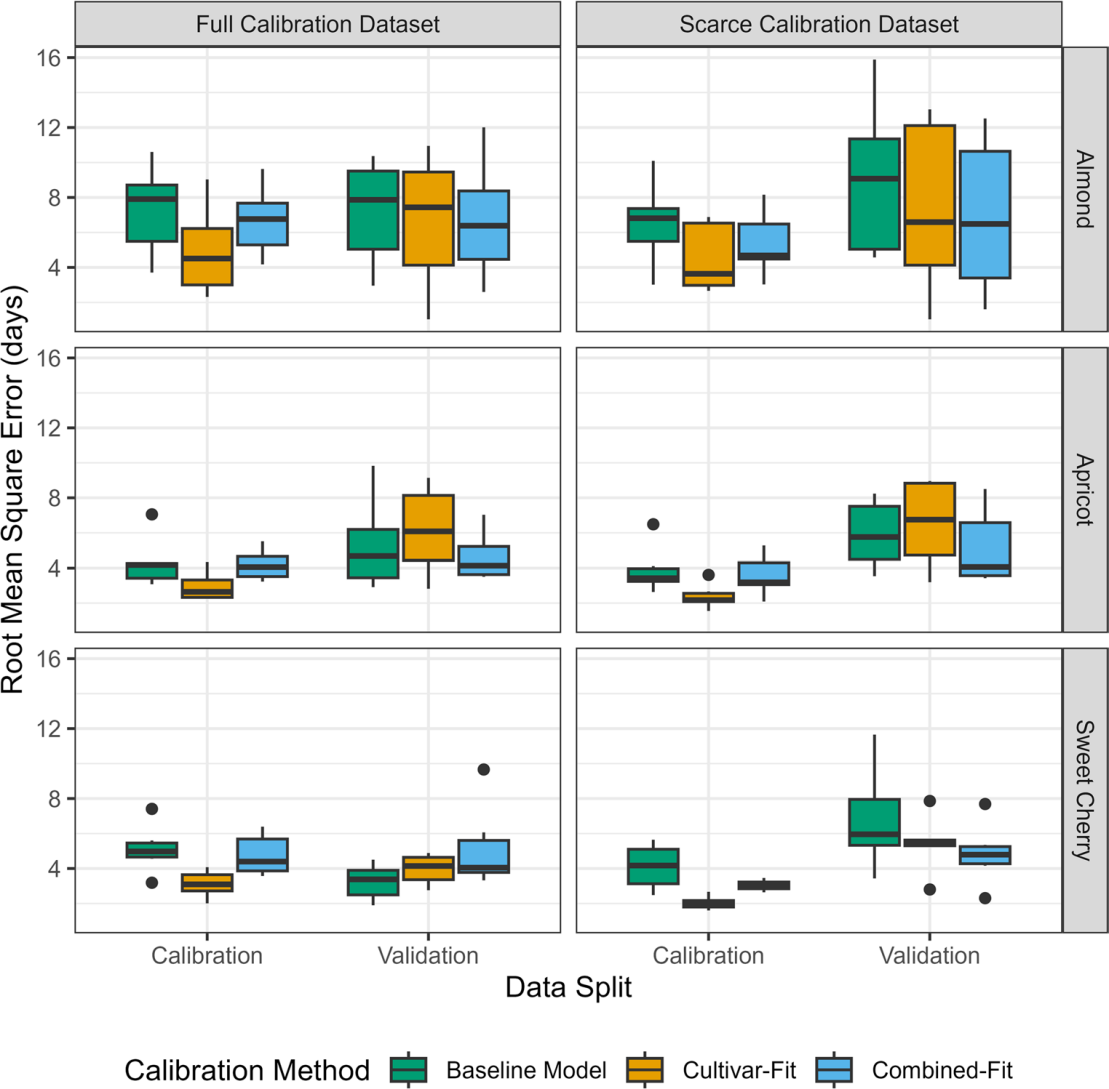
Root mean square error (RMSE) for bloom date prediction using the baseline model with only chill (y_c_) and heat requirement (z_c_) and transition parameter (s_1_) estimated (green boxplots), cultivar-fit models with cultivar-specific chill and heat submodels and requirement parameters (orange boxplots) and combined-fit models with cultivar-specific y_c_, z_c_, s_1_ but species-specific chill and heat submodels (blue boxplots). Model performance was compared among calibration and validation data (x-axis), calibration data size (scarce: 10 observations, full: 75% of observations; indicated by columns) and among species (almond, apricot, sweet cherry, indicated by rows)

**Fig. 4:**
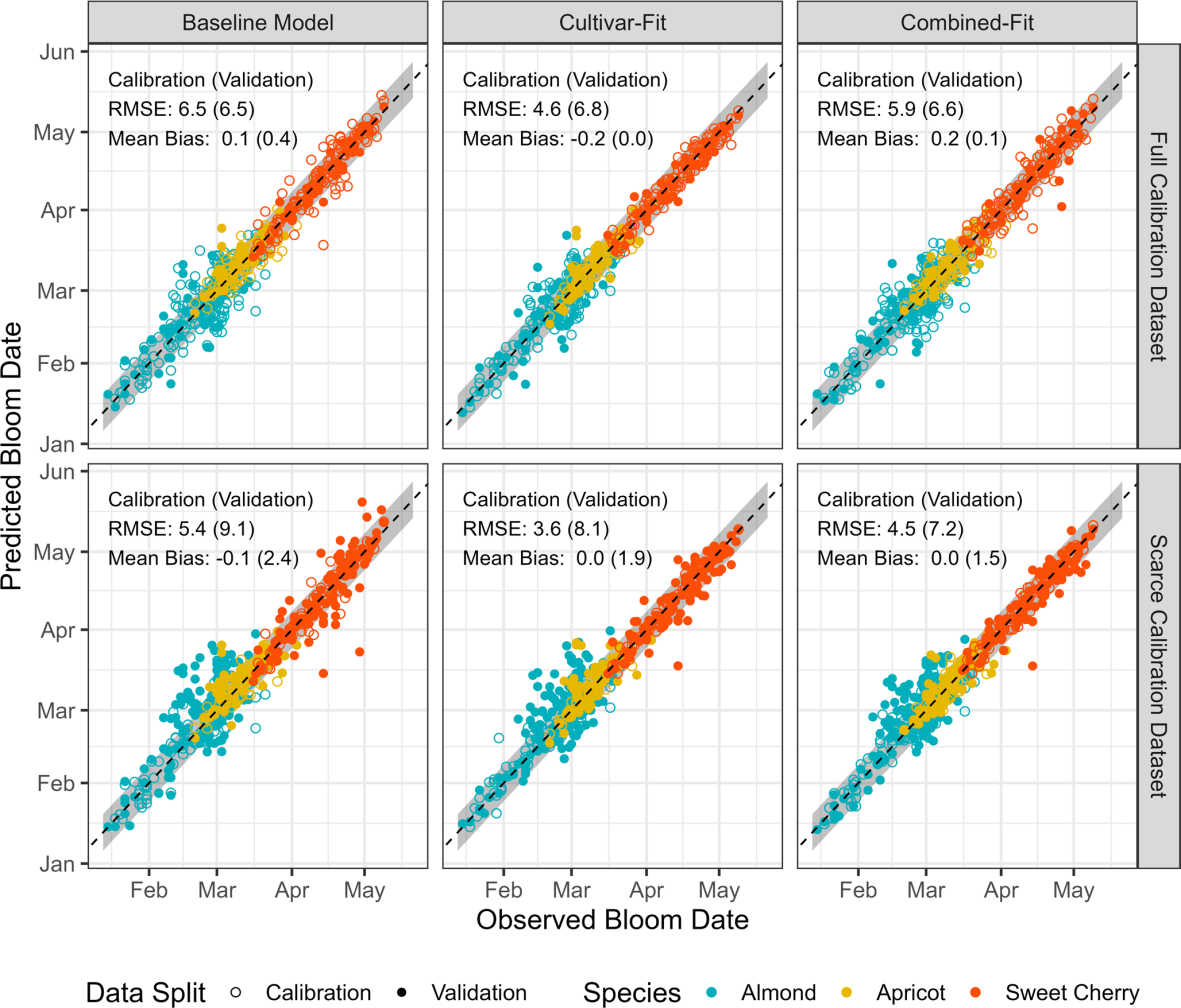
Observed bloom dates (x-axis) and predicted bloom dates (y-axis) for almond (blue dots), apricot (yellow dots) and sweet cherry (red dots). Data split is indicated by dot type: open dots for calibration data and filled dots for validation data. Calibration methods (baseline model, cultivar-fit, combined-fit) are indicated by columns, calibration dataset sizes (full = 75% of all observations, scarce = 10 observations) are indicated by rows. Perfect agreement of prediction and observation is indicated by diagonal dashed line. Predictions errors falling within a range of ± one week are indicated by the shaded grey area. Model performance is indicated by root mean square error (RMSE) and mean bias (predicted minus observed)

When predicting the bloom dates of the calibration dataset, we observed lower RMSE values for cultivar-fit models than for combined-fit and baseline models (Fig. 3). For example, when evaluating models trained on the Full_cal_ almond data, the median RMSE ± standard deviation for calibration data was 4.5 ± 2.2 days for cultivar-fit models, compared to 6.8 ± 1.8 days for combined-fit models and 7.9 ± 2.4 days for baseline models. When using unseen data for validating models (Full_val_), RMSE values were quite similar across the three calibration methods. For almond models trained on Full_cal_ (full calibration data), the median RMSE for the validation data Full_val_ was 6.4 ± 3.0 days for the combined-fit model, 6.8 ± 1.8 days for the cultivar-fit model, and 7.9 ± 2.7 for the baseline model. For the other species, prediction errors for the Full_val_ data were generally lower, with RMSE values for apricot ranging between 4.1 ± 1.4 days (combined-fit) and 6.1 ± 2.5 days (cultivar-fit), and for sweet cherries between 3.4 ± 1.0 days (baseline model) and 4.1 ± 0.9 days (cultivar-fit models). Generally, we observed lower prediction errors for calibration than for validation data (Figs. 3 and 4).

Models calibrated with scarce datasets Scarce_cal_ exhibited larger differences between calibration and validation performance than models calibrated with the full dataset. Among calibration methods, the largest discrepancy between calibration and validation data performance was observed in cultivar-fit models, ranging from 2.2 days (Full_val_ – Full_cal_) to 4.5 days (Scarce_val_ – Scarce_cal_). For the baseline model, the difference between calibration and validation RMSE values was 0.0 days (full calibration dataset) and 3.7 days (scarce calibration dataset), while for the combined-fit approach, it was 0.7 days (full calibration dataset) and 1.7 days (scarce calibration dataset).

We did not find evidence of an interaction between calibration method (baseline model, cultivar-fit, combined-fit) and calibration dataset size (scarce: 10 observations, full: 75% of observations) in determining model performance. Overall, model accuracy appeared to be primarily driven by species, and more specifically by cultivar (Online Resource 1, Figure S1).

Predictions using models calibrated on scarce datasets tended to be systematically late, with a mean bias ranging from 1.5 days (combined-fit) to 2.4 days (baseline model) for the validation data. In contrast, mean bias was low (< ± 0.2 days) for data of the Full_val_ dataset (Fig. 4). The proportion of large prediction errors (residuals > 7 days) ranged from 6% (cultivar-fit, Scarce_cal_) to 33% (baseline model, Scarce_val_). Approximately two-thirds of these large errors occurred in almond models.

**Fig. 5:**
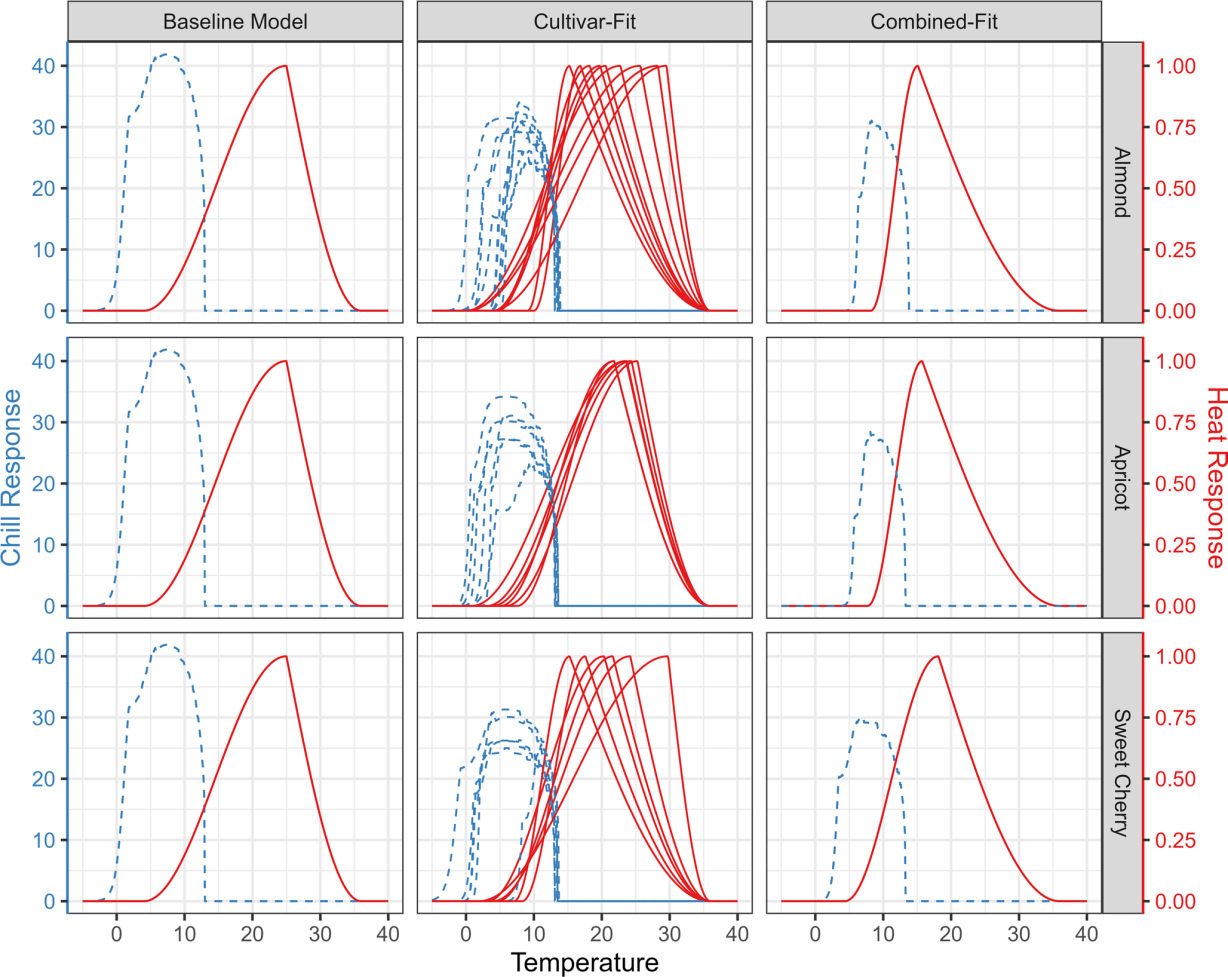
Temperature response curves for chill (dashed blue lines) and heat (solid red lines) submodels of PhenoFlex models calibrated for almond (top row), apricot (middle row) and sweet cherry (bottom row) cultivars using different calibration methods: baseline model (left column), cultivar-fit (middle column) and combined-fit (right column). The response curves visualize how different temperatures contribute to chill and heat accumulation given the estimated model parameters and a period of 1200 h assuming constant temperature. In the baseline model, chill and heat submodel parameters were kept at default values, hence response curves are identical for all species and cultivars. In combined-fit models, chill and heat submodel parameters were shared by cultivars of the same species, therefore one chill and one heat response curve emerge per species. In cultivar-fit models, chill and heat parameters were estimated per cultivar, resulting in a variety of curves per species. Only temperature response curves from the full calibration dataset are shown

For both the chill and the heat submodels, the calibration methods differed in the temperature response curves that are implied by the model parameters (Fig. 5). For the baseline model, a single chill and heat response curve was applied uniformly across all species and cultivars. In contrast, the combined-fit model produced species-specific curves that were identical among cultivars of the same species but differed between species. The cultivar-fit models yielded the highest degree of variation, producing a specific temperature response curve for each cultivar.

 Most chill response curves from cultivar-fit and combined-fit models indicate that chill accumulation begins above freezing temperatures (> 0 °C), while the baseline model also showed sensitivity to sub-zero conditions. For example, chill accumulation starts at approximately 5 °C for almonds and apricots, and around 2 °C for sweet cherry, according to the results of the combined-fit models. Cultivar-fit models for sweet cherry similarly showed chill accumulation starting at lower temperatures than for almonds, although there was substantial variation among cultivars. The chill response curves also differed in peak height, however, these differences were offset by lower or higher estimates of chill requirement.

We also observed variation in the heat response curves. These curves peaked at 1.0 (arbitrary heat unit) by design, precluding comparisons of response strength. However, differences were evident in the starting temperature and optimal temperature for heat accumulation. In cultivar-fit models, both onset and optimal temperature for heat accumulation varied widely, particularly for almond and sweet cherry cultivars. Apricot cultivars showed less variation. In combined-fit models, the onset temperature for heat accumulation was generally higher for almonds and apricots than for sweet cherry. The optimal temperature for heat accumulation was lower in combined-fit models than in the baseline model for all three species. The critical temperature threshold (36 °C) was fixed across all models.

## Discussion

### Similar model performance across calibration methods and dataset sizes

We evaluated different calibration methods for the PhenoFlex phenology model, which varied in specificity to species and cultivars. In all approaches, chill and heat requirements and the transition parameter were cultivar-specific, but the chill and heat submodels differed. The baseline model used default settings, the combined-fit model estimated submodels at the species level, and the cultivar-fit model tailored the submodels to each cultivar. Consequently, the number of estimated parameters varied: three for the baseline model, three cultivar-specific plus seven shared parameters for the combined-fit, and ten for each cultivar-fit model.

We observed that the cultivar-fit approach produced lower, though not drastically lower, calibration RMSEs compared to the combined-fit and baseline models, but similar RMSE values for the validation data (Figs. 3 and 4). Variation in model performance among cultivars was larger than the variation across calibration methods. Contrary to our expectations, the number of estimated parameters did not strongly influence model performance, even with limited calibration data. Previous studies indicated potential overfitting of PhenoFlex to calibration data (Tang et al. 2024), particularly with limited observations. However, our results did not show strong dependency on the size of the calibration dataset. A possible reason may be that our calibration approach did not rely on multiple iterations, as has been the case in other studies (Chuine 2000; Fernandez et al. 2022; Luedeling et al. 2021), so it may have been less prone to overfitting.

Previous studies have reported that fixing chill and heat submodels to default settings led to higher prediction errors compared to a full calibration of the PhenoFlex model (Luedeling et al. 2021). In contrast, we observed negligible differences in model performance when comparing the baseline model with standard chill and heat submodels to customized submodels within cultivar-fit and combined-fit models (Figs. 3 and 4). The differences in prediction accuracy among calibration methods are dwarfed by the general variation in prediction accuracy between cultivars and species. Model performance differences across species were also noted, with almonds showing greater variability than the other species. We suspect, however, that the greater variability in the performance of almond models may have been caused by the almond data originating from climatic conditions that were marginal for the species rather than reflecting inherent differences between the species (Caspersen et al. 2025b). Earlier research (Caspersen et al. 2024) indicated that PhenoFlex can accurately predict almond bloom, suggesting that model performance should not be attributed solely to species.

The combined-fit method presents a viable alternative to cultivar-fitting. Both methods performed similarly, even under data-scarce conditions. However, the combined fitting ensures consistency in chill and heat submodels across cultivars (Fig. 5). In contrast, single-fit models produce distinct submodels per cultivar, complicating comparisons of agroclimatic requirements (e.g., Chill Portions, Growing Degree Hours) across cultivars and hindering model integration into broader agroclimatic studies (Benmoussa et al. 2020; Fernandez et al. 2021a, 2023; Shinwari et al. 2024). Furthermore, species-specific submodels may enhance model transferability to other cultivars of the same species as the number of competing parameter sets is reduced.

### Temperature response curves raise questions

We observed differences in the temperature response among methods (cultivar-fit, combined-fit), among species and, for the cultivar-fit models, also among the cultivars of the same species. In an earlier study, equally well-performing models for pear cultivar ‘Alexander Lucas’ and apple cultivar ‘Boskoop’ showed very different temperature response curves, with the chill response curve for ‘Boskoop’ being strongly distorted, indicating chill accumulation at very high temperatures (> 20 °C) (Luedeling et al. 2021). A PhenoFlex-based analysis of three almond cultivars in the Central Valley of California also revealed differences in response curves (Caspersen et al. 2024). It is unclear whether these findings imply that response curves are species- or even cultivar-specific, or whether the observed variation is an artefact of the calibration. The variation in temperature response curves could also be caused by model equifinality, a condition describing that many parameter combinations can lead to similar model outcomes. Model equifinality is a common problem when modeling phenology (Wallach et al. 2021; Yang et al. 2023). Customizing chill and heat response curves has proven beneficial when modeling winter dormancy and tree bloom (Egea et al. 2021; Luedeling et al. 2021), but the obtained response curves should be interpreted with caution. The temperature response curves implied by the model parameters can, however, serve as a second validation step next to model accuracy measures (RMSE, RPIQ) and help discard model parameters that perform well but lead to distortions in the modeled processes (Caspersen et al. 2024; Luedeling et al. 2021).

Models that are fitted to observed data by adjusting parameters, rather than using independently measured biological values, can achieve high predictive performance, but they risk misrepresenting the underlying processes (Levins 1966, 1968; Sharp 1990 as cited in Hänninen et al. 2019). This approach, known as inverse modeling, estimates parameters by optimizing model output against training data. To reduce the risk of generating biologically implausible temperature response curves, we constrained the parameter search space by narrowing input parameter ranges and replacing highly uncertain parameters with intermediate ones that have more clearly defined bounds (see also Egea et al. 2021 and Caspersen et al. 2024). This strategy helped prevent unrealistic model behavior.

Even relatively simple models with default chill and heat response curves, such as PLS-based methods (Luedeling and Gassner 2012) or estimates based on standard forcing experiments (Ruiz et al. 2007), have been shown to produce chill and heat requirements that are strongly influenced by the local climate and often fail to generalize across locations. These inconsistencies can be attributed to cultivar plasticity (Delgado et al. 2026), but they also reflect overfitting of model parameters to the specific environmental conditions under which the data were collected. Given that such variation occurs even in models with fewer degrees of freedom and more constrained structures, it is likely that more complex models like PhenoFlex are subject to similar limitations. Therefore, parameter estimates from these models should be interpreted with caution, particularly when comparing results across different regions or attempting to generalize findings beyond the calibration dataset.

### Moving spring phenology modeling of fruit trees forward

In response to the growing number of studies on spring phenology modeling, Hänninen et al. (2019) recently articulated a call for coordinated and targeted experiments in spring phenology and dormancy research. Multi-environment experiments on potted trees (Fernandez et al. 2021b), for example, have been used to shed light on alternative dormancy breaking pathways that appear to be inadequately covered by the current spring phenology models (Fernandez et al. 2022). Controlled temperature experiments on chill accumulation, heat accumulation and their interaction (Sugiura et al. 2024) allow empirical estimation of the effectiveness of different temperatures for chill and heat accumulation. In addition to experiments on trees, experimental evaluations on existing spring phenology models are also needed. Benchmarking studies, similar to the evaluation of crop growth models in the Agricultural Model Intercomparison and Improvement Project (AgMIP) or climate models in the Coupled Model Intercomparison Project (CMIP), are still missing for phenology models. Such comparative analyses could help document model characteristics and expose knowledge gaps.

Aside from the needed phenology model benchmarking, we propose that the question of model equifinality needs to be addressed. The variety of temperature response curves for cultivars of the same species already indicates that there are indeed many parameter combinations that capture the bloom dates equally well. We have no way of knowing how distinct from biological reality the modeled temperature response curves are, because there is no ground truth of the model parameters. This would require extensive experimentation, as done in the work leading up to the Dynamic Model. Even with such experimental data, the range of possible model parameters can be considerable (for reference, see the range in the parameters in Table 1 Fishman et al. (1987b)). To better understand what precision we can expect when calibrating model parameters, controlled experiments with the model are needed. Lending from the ‘Virtual Ecologist’ concept (Zurell et al. 2010), we propose to analyze the model not based on real bloom observations gathered in the orchard, but with synthetic bloom dates. Such calibration experiments based on synthetic data could shed light on problems such as sampling effect when collecting bloom data but also on how retraceable model parameters are.

## Conclusion

We explored several calibration approaches for spring phenology models across datasets of differing length. The prediction accuracy remained comparable across calibration methods. However, our newly proposed method, where some parameters are shared among cultivars of the same species and some are cultivar-specific, offers conceptual advantages. It provides greater consistency in the chill and heat accumulation submodels compared to the large variation in cultivar-fit models and the one-size-fits-all approach of the baseline model.

Our results imply that the combined-fit approach allows calibrating PhenoFlex models even for cultivars with limited phenological data, such as newly bred cultivars or understudied local varieties. As fruit tree flowering studies aim to forecast future trends and identify suitable cultivars, our calibration methods may offer a way to expand the range of cultivars that can be analyzed. By lowering data and parameter requirements, our methods support more inclusive modeling, particularly in regions lacking resources for long-term phenological monitoring.

While overall model performance was satisfactory, our findings revealed considerable variability in how PhenoFlex represents the chill and heat accumulation processes across species and cultivars. Whether these differences reflect biological reality or model artefacts remains uncertain. This uncertainty highlights a pressing need: not only for more empirical data gathered from controlled experiments, but also for a deeper interrogation of the models themselves. Rather than expanding the already crowded ecosystem of spring phenology models, research should prioritize experimental designs informed by existing models. The current bottleneck lies less in model availability and more in the understanding of how well these models capture the underlying processes and how robust these conceptualizations will remain under changing climatic conditions.

## Supplementary Information

Below is the link to the electronic supplementary material.

Supplementary Material 1 (PDF 392 KB)

## Data Availability

The phenology and temperature data is part of the publicly available data repository: Luedeling, E., Caspersen, L., Delgado, A., Egea, J.A., Ruiz, D., Ben Mimoun, M., Benmoussa, H., Ghrab, M., Kodad, O., El Yaacoubi, A., Fadón, E., Rodrigo, J., 2024a. Long-Term Phenology Observations for Temperate Fruit Trees in the Mediterranean Region (and Germany). 10.60507/FK2/MZIELI.

## Code Availability

The code is publicly available as annotated R notebooks hosted via GitHub pages: https://larscaspersen.github.io/combined-fitting-bloom/.

The annotated R code includes:

• Preparation of data: https://larscaspersen.github.io/combined-fitting-bloom/notebooks/01-prepare-phenology-preview.html.

• Model calibration for almond data: https://larscaspersen.github.io/combined-fitting-bloom/notebooks/02-calibrate-almond-preview.html.

• Model calibration for apricot data: https://larscaspersen.github.io/combined-fitting-bloom/notebooks/03-calibrate-apricot-preview.html.

• Model calibration for sweet cherry data: https://larscaspersen.github.io/combined-fitting-bloom/notebooks/04-calibrate-cherry-preview.html.

• Model evaluation and plotting: https://larscaspersen.github.io/combined-fitting-bloom/notebooks/05-make-figures-preview.html.
