# Supplementary material for: Combining temperate fruit tree cultivars to fit spring phenology models
Source: Int J Biometeorol. 2026 Jan 21;70(2):33. doi: 10.1007/s00484-025-03068-2 (PMC12823765; doi:10.1007/s00484-025-03068-2)
Supplement: Supplementary file 1 — Supplementary Material 1 (PDF 392 KB) [file 484_2025_3068_MOESM1_ESM.pdf]

Supplementary Materials to the manuscript:
Combining temperate fruit tree cultivars to fit spring phenology models

International Journal of Biometeorology

Caspersen, Lars[a,*]; Schiffers, Katja[a]; Jarvis-Shean, Katherine[b]; Luedeling, Eike[a]

[a] Department of Horticultural Sciences, Institute of Crop Science and Resource Conservation (INRES), University of Bonn, Auf dem Hügel 6, 53121 Bonn, Germany

[b] University of California, Division of Agriculture and Natural Resources, 70 Cottonwood St, Woodland, CA 95695, USA

Corresponding author: Lars Caspersen (lcaspers@uni-bonn.de)

This document contains supplementary materials for the journal article: *Combining temperate fruit tree cultivar to fit spring phenology models*. It includes additional tables and files that were not part of the main article, as well as the code to replicate the analyses. A companion version of the Supplementary Materials can be found also online following the link: https://larscaspersen.github.io/combined-fitting-bloom/
The online version also contains links to the code that we wrote for the analysis.

The phenology observations analyzed here are part of a long-term phenology dataset (Luedeling et al., 2024a) compiled within the *Adapting Mediterranean Orchards (AdaMedOr)* project. Of the more than 270 cultivars in the dataset, a subset of 110 cultivars has been analyzed by Caspersen et al. (2025) using the PhenoFlex framework (Luedeling et al., 2021), available via the R package *chillR* (Luedeling et al., 2024b). In addition to model calibration, the analysis includes climate change impact projections on future bloom dates.

More than 50% of the cultivars in the dataset were not analyzed because the bloom observations were considered too short for calibration with PhenoFlex. We propose an alternative calibration method, combine-fitting, which reduces the number parameters estimated per cultivar and may allow the joint analysis of cultivars of the same fruit tree species. We evaluate combined-fit approach for three temperate fruit and nut species (almond, apricot, sweet cherry) and compare the results with those from a baseline model and from a common calibration approach in which each cultivar is calibrated separately. We perform the analysis for the full dataset and for an artificially shortened dataset.

Parts of the function that we present in this document are available via the R packages *evalpheno* (Caspersen, 2025a) and *LarsChill* (Caspersen, 2025b). Both packages are currently available via GitHub.

Supplementary Table

Table S1 Overview on the full bloom dataset for almond, apricot and sweet cherry cultivars.

Species	Location	Country	Cultivar	Year Start	Year End	n
Almond	Meknes	Morrocco	Ferragnes	1977	2014	38
	Meknes	Morrocco	Marcona	1977	2014	38
	Meknes	Morrocco	Tuono	1974	2014	41
	Santomera	Spain	Achaak	1997	2019	13
	Santomera	Spain	Desmayo	1997	2022	21
	Santomera	Spain	Marta	2005	2021	14
	Sfax	Tunisia	Fasciuneddu	1981	2015	22
	Sfax	Tunisia	Mazzetto	1981	2015	22
	Sfax	Tunisia	Nonpareil	1981	2016	23
Apricot	Cieza	Spain	Búlida	2003	2022	21
	Cieza	Spain	Dorada	2003	2022	20
	Zaragoza	Spain	Goldrich	1999	2021	21
	Zaragoza	Spain	Harcot	1999	2022	22
	Zaragoza	Spain	Henderson	1999	2021	21
	Zaragoza	Spain	Sunglo	1999	2022	22
Sweet Cherry	Klein-Altendorf	Germany	Burlat	1978	2015	29
	Klein-Altendorf	Germany	Regina	1988	2020	32
	Klein-Altendorf	Germany	Schneiders	1984	2019	32
	Zaragoza	Spain	Rainier	1991	2022	24
	Zaragoza	Spain	Sam	1991	2022	24
	Zaragoza	Spain	Van	1991	2022	24

When calibrating the model, we specified the search space for each model parameter. We substituted the model parameters E_0, E_1, A_0 and A_1 of the chill submodel with intermediate parameters θ^*, θ_c, π_c and τ following Egea et al. (2021) and implemented for PhenoFlex by Caspersen et al. (2024).Additionally, we restricted parameters, so that the E_{10} quotient of the process modeling chill formation and degradation ranges between 1.5 and 3.5, a range said to be realistic in biological systems (Egea et al., 2021, Luedeling et al., 2021). During model calibration, the optimization algorithm ran for 5,000 iterations for baseline model; 30,000 evaluations for single-fit; 50,000 evaluations for combined fit. We chose different total number of evaluations for the calibration methods, to account for varying number of model parameters estimated during each individual calibration step. The optimization algorithm estimates model parameters by minimizing the residual sum of squares (RSS) of predicted and observed bloom dates. In a pre-trial we confirmed that by the end of the total number of model evaluations the RSS converged, indicating that the algorithm fails to find parameters providing better model performance.

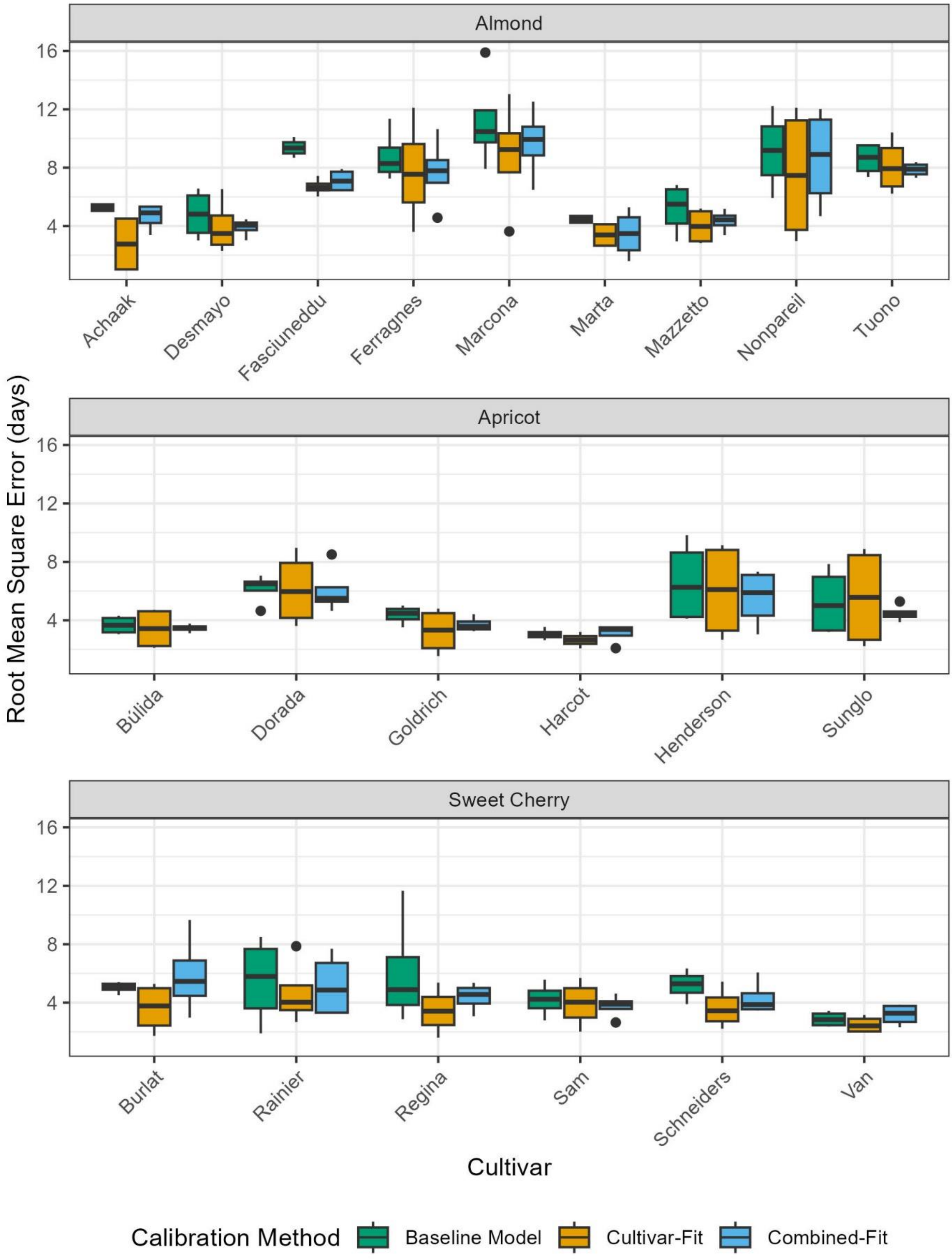

Fig. S1 Root Mean Square Error (RMSE, days) of predicted bloom dates for each species (almond, apricot, sweet cherry) and cultivar (indicated at the x-axis). Boxplot summarizes RMSE for calibration and validation split and for ‘scarce’ and ‘full’ calibration sets. Calibration methods are indicated by color, green for ‘Baseline Model’, yellow for ‘Cultivar-Fit’ and blue for 'Combined-Fit’.

Supplementary Code

The code for analyzes can be found in the notebooks linked in the Online Version of the Supplementary Materials: https://larscaspersen.github.io/combined-fitting-bloom/

The code is documented in notebooks, that also include comments. There are five notebooks in total:

1. **Preparing phenology data.** The data is split into calibration and validation. The split was performed for the full data split and for an artificially shortened version. https://larscaspersen.github.io/combined-fitting-bloom/notebooks/01-prepare-phenology-preview.html

2. **Calibrating almond model.** The notebook covers the three calibration treatments (cultivar-fit, combined-fit and baseline model). The notebook also is commented. https://larscaspersen.github.io/combined-fitting-bloom/notebooks/02-calibrate-almond-preview.html

3. **Calibrating apricot model**. Same as almond but for apricot data. The code is uncommented. https://larscaspersen.github.io/combined-fitting-bloom/notebooks/03-calibrate-apricot-preview.html

4. **Calibrating sweet cherry model**. Same as almond but for sweet cherry data. The code is uncommented. https://larscaspersen.github.io/combined-fitting-bloom/notebooks/04-calibrate-cherry-preview.html

5. **Model evaluation and plotting**. Creates dotplots of predicted and observed bloom dates, boxplot with model RMSE, as well as temperature response plots illustrating how the parameterization of the chill and heat submodel translate into temperature sensitivity of the submodel. https://larscaspersen.github.io/combined-fitting-bloom/notebooks/05-make-figures-preview.html

References

Caspersen, L. (2025a). *evalpheno: Wrapper functions to customize calibration of the PhenoFlex phenology model* (Version v0.0.1) [Computer software]. Zenodo. https://zenodo.org/doi/10.5281/zenodo.15174551

Caspersen, L. (2025b). *LarsChill: Supplementary functions to the dormancy and phenology R-package chillR* (Version v0.0.2) [Computer software]. Zenodo. https://zenodo.org/doi/10.5281/zenodo.15174333

Caspersen, L., Jarvis-Shean, K., & Luedeling, E. (2024). Projecting almond bloom dates in California with the PhenoFlex framework. *Acta Horticulturae*, *1406*, 455–464. https://doi.org/10.17660/ActaHortic.2024.1406.64

Caspersen, L., Schiffers, K., Picornell, A., Egea, J. A., Delgado, A., El Yaacoubi, A., Benmoussa, H., Rodrigo, J., Fadón, E., Ben Mimoun, M., Ghrab, M., Kodad, O., Ruiz, D., & Luedeling, E. (2025). Contrasting Responses to Climate Change – Predicting Bloom of Major Temperate Fruit Tree Species in the Mediterranean Region and Central Europe. *Agricultural and Forest Meteorology*, *375*, 110859. https://doi.org/10.1016/j.agrformet.2025.110859

Egea, J. A., Egea, J., & Ruiz, D. (2021). Reducing the uncertainty on chilling requirements for endodormancy breaking of temperate fruits by data-based parameter estimation of the dynamic model: A test case in apricot. *Tree Physiology*, *41*(4), 644–656. https://doi.org/10.1093/treephys/tpaa054

Luedeling, E., Caspersen, L., Delgado Delgado, A., Egea, J. A., Ruiz, D., Ben Mimoun, M., Benmoussa, H., Ghrab, M., Kodad, O., El Yaacoubi, A., Fadón, E., & Rodrigo, J. (2024). *Long-Term Phenology Observations for Temperate Fruit Trees in the Mediterranean Region (and Germany): A Dataset Compiled by the Adamedor Project* (Version 2.0). bonndata. https://doi.org/10.60507/FK2/MZIELI

Luedeling, E., Caspersen, L., & Fernandez, E. (2024). *chillR: Statistical methods for phenology analysis in temperate fruit trees* [Computer software]. https://cran.r-project.org/web/packages/chillR/. https://cran.r-project.org/web/packages/chillR/index.html

Luedeling, E., Schiffers, K., Fohrmann, T., & Urbach, C. (2021). Phenoflex—An Integrated Model to Predict Spring Phenology in Temperate Fruit Trees. *Agricultural and Forest Meteorology*, *307*, 108491. https://doi.org/10.1016/j.agrformet.2021.108491
